# Clinical trials in cancer: the role of surrogate patients in defining what constitutes an ethically acceptable clinical experiment.

**DOI:** 10.1038/bjc.1989.78

**Published:** 1989-03

**Authors:** W. J. Mackillop, M. J. Palmer, B. O'Sullivan, G. K. Ward, R. Steele, G. Dotsikas

**Affiliations:** Ontario Cancer Foundation, Kingston Regional Cancer Centre, Department of Oncology, Toronto, Canada.

## Abstract

Doctors who treat lung cancer in Ontario were previously asked how they would wish to be managed if they developed non-small cell lung cancer and whether they would consent to participate in six clinical trials for which they might be eligible. The proportion of these expert surrogate patients who would consent to each clinical trial ranged from 11 to 64%. The results of this study were transmitted to the same group of doctors who were asked to comment on the ethical acceptability of each trial in the light of this information. The majority of physicians said that those trials to which less than 50% of expert surrogates consented should not have been opened to patients. Sixty-nine per cent of doctors thought that new trials should be evaluated in this way. We also present the results of a survey of 400 lay people in Ontario who were asked to imagine that they had lung cancer and whether they would consent to participate in two of these same clinical trials. Fifty per cent of lay people consented to a randomised trial of lobectomy versus segmentectomy in early, operable disease (LCSC-821) compared to 64% of expert surrogates, and 48% of lay people consented to a randomised trial of five different forms of chemotherapy in metastatic disease (SWOG-8241) compared to 19% of doctors. It was concluded that the lay people were unable to discern differences in the acceptability of clinical trials which were clear to experts in the field. Subsequently, respondents were told about the decisions which doctors would make in the same circumstances and asked if this information would modify their previous decisions. There is no net change in the proportion of patients consenting to the surgery trial but the proportion of people consenting to the chemotherapy trial decreased by 40%. The majority of lay people said that they would wish to have access to this type of information before consenting to participate in a clinical trial.


Br. J. Cancer (1989), 59, 388-395 © The Macmillan Press Ltd., 1989

### Clinical trials in cancer: the role of surrogate patients in defining what constitutes an ethically acceptable clinical experiment

W.J. Mackillop[1], M.J. Palmer[1,2], B. O'Sullivan[3], G.K. Ward[1], R. Steele[2] & G. Dotsikas[1]

*Ontario Cancer Foundation, Kingston Regional Cancer Centre, Departments of [1]Oncology and [2]Community Health and Epidemiology, Queen's University, Kingston and the [3]Ontario Cancer Institute, Toronto, Canada.*

**Summary** Doctors who treat lung cancer in Ontario were previously asked how they would wish to be managed if they developed non-small cell lung cancer and whether they would consent to participate in six clinical trials for which they might be eligible. The proportion of these expert surrogate patients who would consent to each clinical trial ranged from 11 to 64%. The results of this study were transmitted to the same group of doctors who were asked to comment on the ethical acceptability of each trial in the light of this information. The majority of physicians said that those trials to which less than 50% of expert surrogates consented should not have been opened to patients. Sixty-nine per cent of doctors thought that new trials should be evaluated in this way. We also present the results of a survey of 400 lay people in Ontario who were asked to imagine that they had lung cancer and whether they would consent to participate in two of these same clinical trials. Fifty per cent of lay people consented to a randomised trial of lobectomy versus segmentectomy in early, operable disease (LCSC-821) compared to 64% of expert surrogates, and 48% of lay people consented to a randomised trial of five different forms of chemotherapy in metastatic disease (SWOG-8241) compared to 19% of doctors. It was concluded that the lay people were unable to discern differences in the acceptability of clinical trials which were clear to experts in the field. Subsequently, respondents were told about the decisions which doctors would make in the same circumstances and asked if this information would modify their previous decisions. There is no net change in the proportion of patients consenting to the surgery trial but the proportion of people consenting to the chemotherapy trial decreased by 40%. The majority of lay people said that they would wish to have access to this type of information before consenting to participate in a clinical trial.

Clinical research is necessary to progress in medicine and it is, therefore, essential that we learn to integrate clinical trials into routine practice. This is particularly true of oncology, where only the systemic evaluation of new treatments prevents the widespread use of unproven and often very toxic therapies (Eisenhauer & Mackillop, 1988). When doctors become researchers, however, patients become potential research subjects and this alters the traditional doctor–patient relationship. The clinician-investigator has a dual role as physician in the service of today's patients and scientist in the service of future generations, and with this role comes a potential conflict of interest (Mackillop & Johnston, 1986). The medical profession has recognised this problem and has developed strategies designed to protect both the patient and the doctor in this complex new relationship. The most important of these is the doctrine of 'informed consent'.

The Nuremberg code (1949) stated that the voluntary consent of the subject was absolutely essential in human experimentation and the declaration of Helsinki (World Medical Association, 1964) demanded that 'the potential subject must be adequately informed of the aims, anticipated benefits and potential hazards of the study and the discomfort it may entail'. Hence 'informed consent' became widely accepted as essential in clinical trials but has proved difficult to define and, once defined, difficult to achieve in practice.

Several empirical studies have shown that patients who have given their 'informed consent' for treatment or investigation may have little understanding of what they have consented to (Epstein & Lasagna, 1969; Robinson & Merav, 1976; Schultz *et al.*, 1975; Muss *et al.*, 1979). It has been suggested that one of the barriers to communication is the mental state of the patient with a major illness who may be so emotionally disturbed as to preclude any rational consideration of a proposed clinical trial (Fost, 1975). Furthermore, medical information may not be of value to individuals who do not have the education or experience to understand its significance (Ingelfinger, 1972). We have recently shown that although cancer patients in Canada today usually know their diagnosis, they are often unaware of their prognosis and frequently overestimate the potential benefit of treatment which they are receiving (Mackillop *et al.*, 1988*b*). Thus the patient's right to make the final decision about participating in a clinical trial may be of limited value and the medical profession must retain the responsibility for ensuring that patients are not asked to accept experimental treatments with unacceptably high risk/benefit ratios.

We have developed an approach to this problem of establishing what constitutes an ethically acceptable experiment, based on the 'golden rule' that we should not do anything to other people that we would not want them to do to us. Our strategy is to get disinterested experts to ask themselves if they would consent to participate in a trial if they were in the patient's position. We recently asked doctors who treat pulmonary neoplasms in Ontario to imagine that they had lung cancer (four clinical situations were described) and asked them if they would consent to participate as patient-subjects in six different clinical trials for which they might then have been eligible. The proportion of doctors who would consent ranged from 64% for the most acceptable trial to 11% for the least acceptable trial. Reasons given for refusal varied, but many doctors felt that certain trials offered unacceptable options for treatment. We concluded that some patients with lung cancer currently receive experimental therapies with high risk/benefit ratios which experts in the field would not accept for themselves (Mackillop *et al.*, 1986).

We suggested that new clinical trials should be subjected to evaluation by impartial experts using the surrogates process. The opinions of expert surrogates might be useful to the ethics committees or institutional review boards which must decide what constitutes an ethically acceptable clinical experiment. These data would only be of value, however, if there was agreement among professionals and lay people that the process was valid and produced meaningful results.

We have now resubmitted the results of our lung cancer study to the 79 Ontario doctors who participated in the first

Correspondence: W.J. Mackillop, University of Edinburgh, Dept. of Clinical Oncology, Western General Hospital, Edinburgh EH4 2XU, UK.
Received 4 May 1988, and in revised form, 25 October 1988.

survey to obtain their views on the relevance of these data and to enlist their assistance in interpretation. We also present the results of a survey of 400 lay people in Ontario who were asked: (a) if they would be prepared to participate in two of the clinical trials which we had asked the doctors about; (b) whether the opinions of expert surrogates would influence their decisions; (c) whether they thought that the opinions of expert surrogates should be used in evaluating clinical trials before they were opened to patients; and (d) how these data should be interpreted. We also describe how a number of demographic factors influenced lay people's decisions and how the construction of the questionnaire itself influenced results.

### Methods

*Resurvey of doctors who treat lung cancer*

Each doctor who responded to the original survey was sent a summary of its results together with a second questionnaire (copies of the instruments used in this study are available on request) which asked:

1. Were the studies chosen, in your opinion, appropriately representative of current trials in the four situations?
2. If the information now presented to you had been available before you answered the original questionnaire, would any of your responses about preferred management, or willingness to participate in trials, have changed? If so, in what way?
3. If the information now presented to you had been available *before* these trials were opened to patients should any of them have been stopped? If so, which?
4. Do you think it would be useful to submit *new* clinical trials to expert surrogates in this way before they are opened to patients?

*Survey of lay people*

*The clinical scenarios.* The two clinical situations and the two clinical trials about which we asked the lay surrogates were identical to those which had been used in our previous expert surrogate survey (Mackillop *et al.*, 1986). The two trials chosen were LCSG-821, a randomised phase III study of lobectomy versus wedge resection in early operable non-small cell lung cancer, and SWOG-8241, a randomised phase III study of five different forms of combination chemotherapy in metastatic non-small cell lung cancer. The descriptions of the clinical situations were rewritten avoiding medical terminology and expanded to include an explanation of the nature of the illness, the forms of treatment available, the therapeutic intent and the prognosis. The descriptions of the clinical trials were expanded to include a description of the purpose of each trial and the nature of randomisation. The new draft descriptions of the situations were then submitted to a group of oncologists who were asked to ensure that the description of the clinical situation would be comprehensible to the average patient and that the clinical trials had been fairly described. A second draft incorporating their suggestions was then submitted to a group of 15 lay people from diverse backgrounds. They were asked to complete the form as far as possible and to identify parts which they did not understand or points where they would have liked more information. Further corrections were made and a final draft was then submitted to another group, unconnected with the medical profession, who all found the form comprehensible and were unable to make any further constructive suggestions. The two scenarios are shown, in their final form, in Appendices 1 and 2.

*Description of doctors' attitudes to the clinical trials.* The opinions of doctors about these two clinical trials were known from our previous study of the role of expert surrogates in the evaluation of clinical trials (Mackillop *et al.*, 1986). In their complete form, the doctor's opinions were described as shown in Appendices 3 and 4.

*Questionnaire construction.* The questionnaire in its final form consisted of two sections. In section I respondents were asked to imagine that they had been found to have lung cancer. They were asked to consider the two clinical situations in detail and to decide if they would be prepared to consent to participate as patient–subjects in the two clinical trials. They were asked to give reasons for their decisions. When this section of the form had been completed, the respondents opened a sealed envelope which contained a description of the decisions which doctors believed they would make in the same situations. In section II of the questionnaire the respondents were then asked to review their previous decision and to decide how they would wish to act in the light of this new information. If they changed their minds, they were asked to explain why and, if not, they were asked to explain why not.

*Variations in questionnaire structure.* We wished to determine whether the manner in which the doctors' decision was presented influenced lay people's decisions and whether the inclusion of the doctor's reasons for refusing to participate would have an effect on lay people's decisions independent of the simple proportion of doctors who would consent. Thus, the section of the questionnaire presenting the doctors' reasons finally had five different versions, each of which was submitted to one-fifth of the sample population. The first group received only a statement of the percentage of doctors who would consent to participate (positive frame), while the second group received only a statement of the percentage of doctors who would not consent to participate (negative frame). The third group received a statement of the percentage of doctors who would consent and the percentage of doctors who would not consent to participate (mixed frame). The fourth group received a statement of the percentage of doctors who would not consent to participate and a summary of their reasons for refusing to participate (negative frame and reasons), and the fifth group received a statement of the proportion of doctors who would consent, the percentage of doctors who would not consent and the reasons given by those who refused to participate (mixed frame and reasons). Doctors' reasons for consenting were not elicited in the previous study and could not, therefore, be included in this section.

*Information about the population surveyed.* The questionnaire included a section requesting the following basic information about the subject: age, sex, country of birth, marital status, occupation, educational level and smoking status. We also determined whether the subject had ever had cancer or any other major illness. Each subject was asked about personal experience of cancer and of the various forms of cancer treatment in close friends or relatives.

*Recruitment of research subjects.* No attempt was made to obtain a random sample of Ontario society. We reasoned that only highly motivated subjects would be willing to complete this rather difficult questionnaire and that we could not expect more than a very low compliance if we selected subjects at random. Eighty volunteers (40 in Toronto and 40 in Kingston) each agreed to distribute and collect five copies of the questionnaire among their acquaintances. The Kingston recruiters were 40 members of the scientific and non-medical staff of the Kingston Regional Cancer Centre, and in Toronto 20 recruiters were volunteers from the Canadian Cancer Society and 20 were paramedical staff from the Princess Margaret Hospital. The recruiters were asked to distribute their questionnaires to people who were over the age of 20, who were not physicians and who were not, themselves, cancer patients. The recruiters were instructed not to discuss the questionnaire with the volunteers until after it had been returned completed. Volunteers were told that the questionnaire concerned public attitudes to cancer research and treatment and that the questionnaire would take at least half an hour to complete. They were asked not to accept the questionnaire unless they believed they would find the time to complete it. The recruiters were encouraged to try and get the questionnaires back within two weeks and all those which were analysed were returned within one month. The recruiters told each of their volunteers that the sealed package which they were given contained a questionnaire and an inner sealed envelope which must not be opened until the first section had been completed. Volunteers were asked to complete the questionnaire alone and in private and not to discuss it with others until it had been completed.

*Data management and analysis.* A dBase III Plus (Ashton-Tate) file was written to accommodate the data, which were primarily abstracted from the questionnaire on to an abstraction sheet and subsequently entered into the computer using a screen format identical to that of the abstraction sheet. The information was then uploaded to the Queen's University mainframe computer for analysis by Statistical Analysis System (SAS) version 5 (SAS Institute Inc.). $\chi^2$ contingency table analysis was used to study the significance of simple differences in proportions and multifactor analysis (logistic regression) was used to study the influence of multiple variables on decisions regarding individuals' consent to participate in clinical trials.

### Results

*Doctors' interpretation of expert surrogate data*

Only 45 of the 79 doctors (57%) completed the second questionnaire. Of these, 84% thought that the six trials chosen in the first study were representative of ongoing work in the field. Ninety-three per cent said that the views of their colleagues would not alter their personal decisions regarding their choice of management or their decision to participate or not in the clinical trials. Sixty-nine per cent of our respondents thought that new clinical trials should be submitted for evaluation by expert surrogates before they were opened to patients.

Table I shows that when the majority of expert surrogates consented to a given trial there was almost unanimous agreement among doctors that the trial was acceptable for patients. Even those doctors who did not personally consent to participate agreed that these trials were acceptable. On the other hand, where most doctors did not consent, the majority of those who would personally refuse regarded the trial as unacceptable but the majority of those who would personally participate regarded the trial as acceptable for patients.

*Survey of lay people*

*Characteristics of the population surveyed.* Three hundred and forty-four of the 400 questionnaires (86%) were returned appropriately completed. Two-thirds of the respondents were women. Thirty-one per cent were 19–29 years old, 23% were 30–39, 18% were 40–49 and 28% were 50 or older. One-half resided in Toronto, and one-half resided in Kingston. (The responses of people from Toronto did not differ from the responses of people from Kingston, so the entire group of respondents is considered together.) Most individuals were married (57%). Nearly one-half (47%) had a university education, one-quarter (26%) had a college education and one-quarter (27%) had either an elementary or high-school education. The majority (79%) were born in North America. Seventy-eight per cent were non-smokers. Table II illustrates that the majority of respondents were in good health and only a minority had experienced a major illness in their lifetime. Most, however, were familiar with cancer through the experiences of close friends or relatives.

*Attitudes of surrogate patients to the surgical trial (LCSG-821).* Surrogate patients were asked if they would participate in the surgical trial outlined above (LCSG-821) and 343 answered this question. One hundred and seventy-three (50.4%) consented and 170 (49.6%) refused. Reasons given

**Table II** Experience of illness

| *Experience* | *%* |
|---|---|
| Serious illness requiring hospitalisation | 21.0 |
| Chronic medical illness | 14.0 |
| Malignant disease | 1.5 |
| Close friend/relative with cancer | 83.0 |
| Close friend/relative who received radiotherapy for cancer | 61.0 |
| Close friend/relative who received chemotherapy for cancer | 58.0 |
| Close friend/relative who received surgery for cancer | 68.0 |
| Close friend/relative who died of cancer | 68.0 |

**Table III** Reasons to participate or not to participate in the surgery study

| | |
|---|---|
| *Reasons to participate* | |
| Perceived absence of risk | 47.4% |
| Altruism | 41.6% |
| Expectation of best possible care on study | 15.6% |
| Preference for experimental treatment | 12.7% |
| Trust in doctors | 5.2% |
| *Reasons not to participate* | |
| Preference for standard treatment | 61.8% |
| Objection to randomisation | 27.1% |
| Lack of information | 14.1% |
| Preference for experimental treatment | 7.1% |
| Preference for personalised treatment | 6.5% |

Often more than one answer was given.

**Table I**

| *Study codename* | *Percentage consent by expert surrogates in original survey* | *Percentage of doctors who said the trials should be stopped in the resurvey* | | |
|---|---|---|---|---|
| | | *All doctors* | *Doctors who personally consented* | *Doctors who personally refused* |
| LSCG-821 | 64 | 5 | 4 | 8 |
| EORTC-08824 | 57 | 0 | 0 | 0 |
| LCSG-791 | 31 | 41 | 20 | 46 |
| YALE-LUN-1 | 27 | 53 | 16 | 59 |
| SWOG-8241 | 19 | 59 | 29 | 65 |
| NCCTG-812451 | 11 | 55 | 25 | 57 |

for consent or for refusal are shown in Table III. The most common reason given for participating in this trial was the view that it presented no risk but many also stated that they would participate in order to help other people with cancer in the future. A few believed that they would receive the best possible care by participating in the clinical trial. The most common reason given for refusing to participate was a preference for the standard treatment. Only a small minority expressed a preference for the experimental treatment. In addition, a substantial number expressed a specific objection to the process of randomisation in this setting. A few felt that insufficient information about the trial had been given to allow them to participate.

After the surrogates had been informed of the doctors' decisions in the same circumstances, they were asked again if they would consent to participate in this clinical study and 49.6% now consented and 50.4% refused. Fifteen people had changed their decision from yes to no and 13 from no to yes.

Figure 1 shows the effect of the subject's age on the frequency of consent to the trial, both before and after being informed of the decisions of the doctors. People under 30 and over 60 were willing to consent to the trial more frequently than the intermediate age groups ($P<0.05$). Participation was not influenced by sex, education, marital status, country of birth, experience with cancer or smoking status.

Table IV shows the way in which respondents' decisions were influenced by the doctors' opinions according to the type of information which the subject had received. There was a significantly higher frequency of transitions from yes to no in the subgroup which had received the information about the doctors' decisions in the negative frame accompanied by a statement of their reasons for refusing ($P<0.05$). The presentation of the information in the negative frame alone without giving the doctors' reasons for refusal did not produce this effect nor did the addition of the reasons when the doctors' views were presented in the mixed frame (for an explanation of positive, negative and mixed frame see **Methods**).

*Attitudes of surrogate patients to the chemotherapy trial (SWOG-8241).* Surrogate patients were asked if they would participate in the chemotherapy trial outlined above and 341 answered this question. One hundred and sixty-four (48.1%) consented and 177 (51.9%) refused. Reasons given for consent or refusal are shown in Table V. The most common reason for consent to this trial was the belief that participation would help others in the same situation in the future. A large proportion of patients also believed that they would receive the best possible care by participating in the study. In this situation, only a few patients stated that they could see no risk in participation. The most common reason for refusing to participate was concern about the toxicity of the treatment or the perceived poor quality of life associated with the forms of treatment involved in the study. A large proportion also expressed the concern that the chemotherapy was not likely to help them very much. A few expressed a preference for standard treatment and some felt that insufficient information had been presented to permit them to consent. A smaller proportion objected to randomisation in this context than in the context of the previous study.

After the surrogates had been told of the doctors' decisions in the same circumstances, they were asked again if they would consent to this study and 30.1% now consented and 69.9% refused. Sixty-one individuals changed their mind from yes to no and one from no to yes and this difference is statistically significant ($P<0.0001$).

Figure 2 shows the effect of the age of the subjects on the frequency of consent to the chemotherapy trial. As in the

**Table IV** The frequency of transitions in the surgical trial (LCSG-821) as a function of the way in which the doctors' decisions were communicated to the respondents

| *Frame* | *YN* | *NY* | *YY* | *NN* | *Total* |
|---|---|---|---|---|---|
| Positive | 2 | 5 | 33 | 27 | 67 |
| Negative | 0 | 2 | 31 | 36 | 69 |
| Mixed | 1 | 1 | 32 | 33 | 67 |
| Negative + reasons | 9 | 2 | 23 | 33 | 67 |
| Mixed + reasons | 3 | 3 | 35 | 27 | 68 |
| Total | 15 | 13 | 154 | 156 | 338 |

The framing variables introduced into the questionnaire are described in detail in **Methods**. *YN* = respondents who originally consented to participate in the trial and later refused when informed of the doctors' decisions; *NY* = those who originally refused to participate in the trial and later consented when informed of the doctors' decisions; *YY* = those who consented both before and after being informed of the doctors' decisions; *NN* = those who refused both before and after being informed of the doctors' decisions.

**Table V** Reasons to participate or not in the chemotherapy study

| | |
|---|---|
| *Reasons to participate* | |
| Altruism | 62.8% |
| Expectation of best possible care on study | 61.6% |
| Perceived absence of risk | 13.6% |
| Trust in doctors | 1.8% |
| *Reasons not to participate* | |
| Perception that treatment would result in poor quality of life | 59.3% |
| Perception that chemotherapy is not effective | 47.0% |
| Preference for standard treatment | 13.6% |
| Lack of information | 10.2% |
| Objection to randomisation | 8.5% |
| Perception that the trial was useless | 5.1% |

**Figure 1** The effect of age on the frequency of consent by lay people to participate in the surgical trial (LCSG-821), before and after disclosure of the doctors' decisions. Filled columns, before; hatched columns, after.

**Figure 2** The effect of age on the frequency of consent by lay people to participate in the chemotherapy trial (SWOG-8241), before and after disclosure of the doctors' decisions. Filled columns, before; hatched columns, after.

surgery study, patients over the age of 60 and under the age of 30 were more likely to consent to the trial before receiving any information about the doctors' decisions, but this was not significant. Participation was not influenced by sex, education, marital status, country of birth, experience with cancer or smoking status. Young people, however, were more likely to change their minds when informed of the doctors' decisions than were the elderly ($P<0.05$).

There was no association between consent to the surgery trial and consent to the chemotherapy trial. Forty-six per cent of lay people who consented to the surgery trial also consented to the chemotherapy trial, and 50% of lay people who did not consent to the surgery trial consented to the chemotherapy trial.

Table VI shows the frequency of transitions in response to the doctors' decisions as a function of the type of questionnaire the subject had received. In this situation, neither the manner in which the doctors' views were presented nor inclusion or omission of the reasons for their decisions, influenced the behaviour of the surrogate patients. In each case approximately 40% of those who had initially consented changed their minds.

*Attitudes of lay people to expert surrogate process.* Eighty-three per cent of lay people stated that they would wish to know the views of expert surrogates before deciding whether to consent to a clinical trial if they had cancer and 79% thought that any cancer patient should be given this type of information before being asked to consent to participate in a clinical trial. Most of our subjects (74%) believed that cancer specialists should be asked if they would be willing to participate in clinical trials before these were opened to patients. Those who thought that it was useful to ask cancer specialists this question were also asked what proportion of doctors should be required to consent in order that a trial be considered ethically acceptable. Their answers to this question are expressed in the form of a cumulative frequency distribution in Figure 3.

**Table VI** Frequency of transitions in the chemotherapy trial (SWOG-8241) as a function of the way in which the doctors' decisions were communicated to the respondents

| *Frame* | *YN* | *NY* | *YY* | *NN* | *Total* |
|---|---|---|---|---|---|
| Positive | 13 | 0 | 19 | 33 | 65 |
| Negative | 11 | 0 | 19 | 39 | 69 |
| Mixed | 13 | 1 | 21 | 31 | 66 |
| Negative + reasons | 11 | 0 | 18 | 39 | 68 |
| Mixed + reasons | 13 | 0 | 24 | 32 | 69 |
| Total | 61 | 1 | 101 | 174 | 337 |

The framing variables introduced into the questionnaire are described in detail in **Methods**. *YN* = respondents who originally consented to participate in the trial and later refused when informed of the doctors' decisions; *NY* = those who originally refused to participate in the trial and later consented when informed of the doctors' decisions; *YY* = those who consented both before and after being informed of the doctors' decisions; *NN* = those who refused both before and after being informed of the doctors' decisions.

**Figure 3** The proportion of lay people who regard a clinical trial as acceptable as a function of the proportion of experts who would be prepared to participate in it.

### Discussion

We have shown that lay people were unable to discern any difference in acceptability between two clinical trials which appeared markedly different to experts in the field. Although the individuals surveyed do not represent a cross-section of society, it is improbable that the average lay person would prove to be more discriminating than the members of this unusually well educated group. The surrogate patients also had the opportunity to reflect on the possible costs and benefits of treatment without the emotional stress of a real illness (Fost, 1975). It is, therefore, improbable that the average cancer patient would, in reality, exercise better judgement than these surrogate patients. Modern medical ethics emphasise patients' rights to make their own decisions about their medical care (Sider & Clements, 1985) but lay people appear to be ill-equipped to judge for themselves the risks and benefits of participation in a clinical trial. Some form of review process is, therefore, essential to ensure that patients are not asked to participate in clinical experiments with unacceptable risk/benefit ratios.

The issue of what constitutes an ethical clinical experiment is currently decided by the experts who design clinical trials, who may not be disinterested parties, and by disinterested reviewers on an ethics committee, who may not be experts. We believe that this system of evaluation is flawed by the separation of the two key elements of expertise and impartiality and that additional strategies must be developed to assist in judging the ethical acceptability of clinical research protocols.

The majority of doctors and lay people surveyed in this investigation were in favour of the use of expert surrogates to evaluate new clinical trials before they were opened to patients, although we acknowledge that the sample of professional opinion was less than optimal. There was also a reasonable degree of consensus on how the results of an expert surrogate evaluation might be interpreted. Lay people, on average, wanted the consent of 64% of experts before a trial was opened to patients. Regardless of their personal treatment preference, doctors were almost unanimous that trials to which more than 50% of expert surrogates consented were acceptable. Thus, trials to which two-thirds of expert surrogates consent appear ethically acceptable to the majority of lay people and physicians.

We have previously suggested that, apart from the proportion of doctors consenting, the reasons given by those who refuse should also be carefully considered. If, for example, some doctors refused to participate in a two-arm randomised trial because they had a preference for one treatment option offered in the trial while others refused to participate because they preferred the other option, then their refusal would merely confirm the existence of the controversy which the trial was designed to address. A low overall consent by expert surrogates in this situation could be regarded as a demonstration of the 'clinical equipoise' which Freedman (1987) regards as the hallmark of an ethical randomised trial. However, this concept is not relevant to the two clinical trials which were evaluated here by lay people since we found no evidence of equipoise. In one trial all those experts who refused to participate rejected all arms of the study (SWOG-8241) and in the other all those who refused rejected only the experimental arm (LCSG-821) (Mackillop *et al.*, 1986).

The problem of applying the golden rule to medical decision making is that we run the risk of inflicting our personal system of values on our patients. We cannot assume that our choice of treatment will automatically be correct for a patient, particularly in a palliative setting where optimal treatment may vary according to patients' willingness to take risks and the way in which they weigh quality versus quantity of life. However, it does appear to us reasonable for the medical profession to apply the rule collectively, particularly if it is framed in its negative form, i.e. that a doctor should not offer a patient treatment which the majority of experts in the field would refuse if they were in the patient's position (Mackillop *et al.*, 1988*a*).

The majority of lay people would wish to know, for themselves, whether experts in the field would consent to participate in a clinical trial before they would agree to participate. It has also been demonstrated that the views of expert surrogates may lead potential research subjects to alter their decisions. Thus, the data resulting from an expert surrogate survey might be regarded as material information which should be given to patients who are asked to consent to participate in a clinical trial although it was not our initial intention that the information should be used in this way.

The opinions of lay people have previously been studied in the attempt to determine individual patient preferences for management in a number of situations (McNeil *et al.*, 1982; O'Connor *et al.*, 1985). One important finding was that the manner in which information is presented to lay people has an effect on the way that they interpret it. We have also shown that the manner in which data are presented to surrogate patients may influence their response, particularly when the collective views of the physicians are not uniform. When, however, a large majority of doctors is in favour of one course of action, the manner in which their opinions are presented to surrogate patients has no effect on the way in which they influence surrogate patients' decisions.

Clinical research is undoubtedly an important part of oncology today. Clinical trials have already resolved many long-standing controversies and now ensure that new forms of therapy are evaluated fairly and cautiously before they become accepted into routine practice. It is vitally important, therefore, that the medical community should set high and consistent standards for the practice of clinical research. In the long-term, the number of patients available and willing to participate in clinical experiments will depend on the credibility of the research community in the eyes of the public and of the medical profession as a whole. Further attempts to refine the process of reviewing ethical standards in clinical research are not merely important to protect the interests of today's patients but also to protect the integrity of a process which promises great benefits for future generations of patients.

The authors wish to acknowledge the help of the many volunteers from the non-medical staff of the Ontario Cancer Foundation, Kingston Clinic and Princess Margaret Hospital, Toronto, as well as the members of the Canadian Cancer Society in Toronto who distributed and collected the questionnaires. We are also grateful to our colleagues at the Kingston Regional Cancer Centre for their assistance in the development of the questionnaire. We thank Dr J. Bickenbach of the Faculty of Law at Queen's University for his helpful advice on the structure of the survey and for his valuable criticism of the manuscript. We are indebted to Mrs Stella Greig for her care and patience in the preparation of the manuscript. This work was supported by a grant from the National Cancer Institute of Canada to W.J.M. and R.S.

### Appendices

*Appendix 1*

SITUATION A

*You are found to have lung cancer:*

An X-ray taken of your chest during a routine check-up shows an abnormality in your right lung. You are admitted to hospital for further investigations. A chest specialist passes a tube down your throat and finds a small tumour in your right lung. Further testing confirms a diagnosis of lung cancer. You have no symptoms, and feel fine.

*Your doctor tells you about possible treatments:*

Your doctor explains to you that the right lung has three sections, called lobes, and that your cancer is only in the upper (top) lobe. Surgery is the best method of treating this kind of early lung cancer, and all of the cancer can often be removed by surgery. The usual treatment is to cut out the entire upper lobe. Some doctors think that it should be possible to cure this kind of tumour with a smaller operation, by cutting out a segment of the upper lobe containing the cancer. It is not known which is the better treatment.

*You are asked to participate in a study:*

Your doctor asks you if you would be willing to participate in the study that the doctors in the hospital are involved with to see whether it is better to remove the entire lobe or only a segment of it. All of the patients in the study have lung cancer that can be removed by surgery, like yourself. The intent of the surgery is to cure you of the cancer.

Participation in the study is voluntary. Your doctor tells you that you have every right not to take part. You are assured that if you decide not to take part, you will be treated with surgery in the usual way (complete removal of upper lobe). If you agree to participate, there is an equal chance that you would either have the entire upper lobe of your right lung removed by surgery, or have a segment of it removed by surgery. The treatment that you receive would be decided randomly by a computer. (This means the treatment is decided by chance, just like flipping a coin.) Your doctor does not know in advance which kind of treatment you would receive.

*You are told about possible side effects:*

Your doctor tells you that there is always a slight risk associated with surgery, but the chances of severe complications, or death, are small. There is no known difference between the two operations in terms of risk. Most patients are in the hospital for two or three weeks, and have some pain around their incisions. After spending a month or so recuperating at home, you should feel fine, and only need to return to the hospital for regular checkups after that.

*Appendix 2*

SITUATION B

*You are found to have lung cancer:*

You have generally been feeling unwell for some time, and have lost 20 pounds in weight. You begin to experience a constant pain in your lower back, and make an appointment to see your doctor. A chest X-ray shows an abnormality in your right lung that is suspected of being lung cancer. You are admitted to hospital for further investigations. A chest specialist takes a sample of the tumour and you are told the next day that you have lung cancer. Further testing is carried out over the next few days. Your doctor then tells you that the cancer has spread from the lung to the bones in your lower back and this is why you have pain in that area.

*Your doctor tells you about possible treatments:*

You are told that because the cancer has spread, surgery would be of no use. However, the cancer may be treated by chemotherapy or radiotherapy. There are many different combinations of drugs that could be used. It is not known which is the best combination.

*You are asked to participate in a study:*

Your doctor asks you if you would be willing to participate in the study that the doctors in the hospital are conducting to see which of five possible combinations of drugs is best, in treating a cancer like yours. All of the patients in the study have lung cancer that cannot be removed by surgery, and symptoms like the back pain and weight loss that you have experienced. The intent of the chemotherapy is to shrink the tumour or at least to slow down its growth, but there is no hope of cure.

Participation in the study is voluntary. Your doctor tells you that you have every right not to take part. You are assured that if you decide not to take part, you would be given radiation treatment to the painful areas, and painkillers. If you agree to participate, there is an equal chance that you would receive one of five different kinds of combination chemotherapy as treatment for your lung cancer. Each combination involves two or three drugs. The treatment that you receive would be decided randomly by a computer. (This means the treatment is decided by chance, just like flipping a coin.) Your doctor does not know in advance which kind of treatment you would receive.

*You are told about possible side effects:*

Your doctor tells you that the drugs will be given to you through a tube put into a vein in your arm. The treatment is given every three weeks. Any one of the drug combinations will make you feel sick to your stomach and cause you to vomit for a day or two, and you will also lose your hair. During the course of the treatment, it is possible that you may also experience other side effects such as an increased risk of infection, bruising or bleeding, fever, bladder irritation, numbness and tingling in hands and feet, diarrhoea, and kidney damage. The doctors will make every effort to avoid these more serious complications.

*Appendix 3*

SITUATION A

This is what the Ontario cancer specialists said when we asked them if they would consent to be treated as patients in the first study, in *Situation A*:

64% of the doctors would consent to participate in the study of complete versus partial surgical removal of the upper lobe.

36% of the doctors refused to participate in the study of complete versus partial surgical removal of the upper lobe.

Most of the doctors who refused to participate did so because they had a definite preference for one of the two treatments offered by the study.

33% of the doctors refused to participate because they preferred complete surgical removal of the upper lobe. All of them said that they thought that the smaller operation might be inadequate.

None of the doctors preferred the other form of treatment in which only a segment of the lung is removed.

3% of the doctors would not participate because they wanted additional chemotherapy or radiation treatment, which were not included in the study.

*Appendix 4*

SITUATION B

This is what the Ontario cancer specialists said when we asked them if they would consent to be treated as patients in the second study, in *Situation B*:

19% of doctors would consent to participate in the study of the five types of combination chemotherapy.

81% of doctors refused to participate in the study of the five types of combination chemotherapy.

Most of the doctors refused to participate because they did not want any form of chemotherapy in this situation. Of those doctors who would not participate, 70% thought that chemotherapy would be ineffective and 60% thought that chemotherapy would be too toxic. 17% of doctors wanted radiation as additional treatment.

### References

EISENHAUER, E.E. & MACKILLOP, W.J. (1988). Focus on clinical trials. In *Cost/Benefit Controversies in Cancer Treatment*, Stoll, B. (ed). Macmillan: London.

EPSTEIN, L.C. & LASAGNA, L. (1969). Obtaining informed consent: form or substance. *Arch. Intern. Med.*, **123**, 682.

FOST, N. (1975). A surrogate system for informed consent. *JAMA*, **233**, 800.

FREEDMAN, B. (1987). Equipose and the ethics of clinical research. *N. Engl. J. Med.*, **317**, 141.

INGELFINGER, F.J. (1972). Informed (but uneducated) consent. *N. Engl. J. Med.*, **287**, 465.

MACKILLOP, W.J. & JOHNSTON, P.A. (1986). Ethical problems in clinical trials: the need for empirical studies of the clinical trials process. *J. Chronic Dis.*, **39**, 177.

MACKILLOP, W.J., PALMER, M.J., WARD, G.K. & 4 others (1988*a*). The expert surrogate system: a role for the golden rule in clinical practice and a warning of the dangers. *Humane Med.*, **4**, 89.

MACKILLOP, W.J., STEWART, W.E., GINSBURG, A.D. & STEWART, S.S. (1988*b*). Cancer patients' perceptions of their disease and its treatment. *Br. J. Cancer*, **58**, 355.

MACKILLOP, W.J., WARD, G.K. & O'SULLIVAN, B. (1986). The use of expert surrogates to evaluate clinical trials in non-small cell lung cancer. *Br. J. Cancer*, **54**, 661.

McNEIL, B.J., PAUKER, S.G., SOX, H.C. & TVERSKY, A. (1982). On the elicitation of preferences for alternative therapies. *N. Engl. J. Med.*, **306**, 1259.

MUSS, H.B., WHITE, D.R., MICHIELUTTE, R. & 5 others (1979). Written informed consent in patients with breast cancer. *Cancer*, **43**, 1549.

NUREMBERG TRIBUNAL (1949). Trials of war criminals before the Nuremberg Military Tribunals under Control Council Law, No. 10, Vol. 2, Washington, DC, US Government Printing Office, p. 181.

O'CONNOR, A.M., BOYD, N.F., TRITCHLER, D.L., KRIUKOV, Y., SUTHERLAND, H. & TILL, J.E. (1985). Eliciting preferences for alternative cancer drug treatments: the influence of framing, medium, and rater variables. *Med. Decision Making*, **5**, 453.

ROBINSON, G. & MERAV, A. (1976). Informed consent: recall by patients tested postoperatively. *Ann. Thorac. Surg.*, **22**, 209.

SCHULTZ, A.L., PARDEE, G.P. & ENSINCK, J.W. (1975). Are research subjects really informed? *West J. Med.*, **123**, 76.

SIDER, R.C. & CLEMENTS, C.D. (1985). The new medical ethics. A second opinion (editorial). *Arch. Intern. Med.*, **145**, 2169.

WORLD MEDICAL ASSOCIATION (1964). Recommendations guiding medical doctors in biomedical research involving human subjects, adopted by the 18th World Medical Assembly, Helsinki, Finland, 1964, and revised by the 29th World Medical Assembly, Tokyo, Japan, 1975.


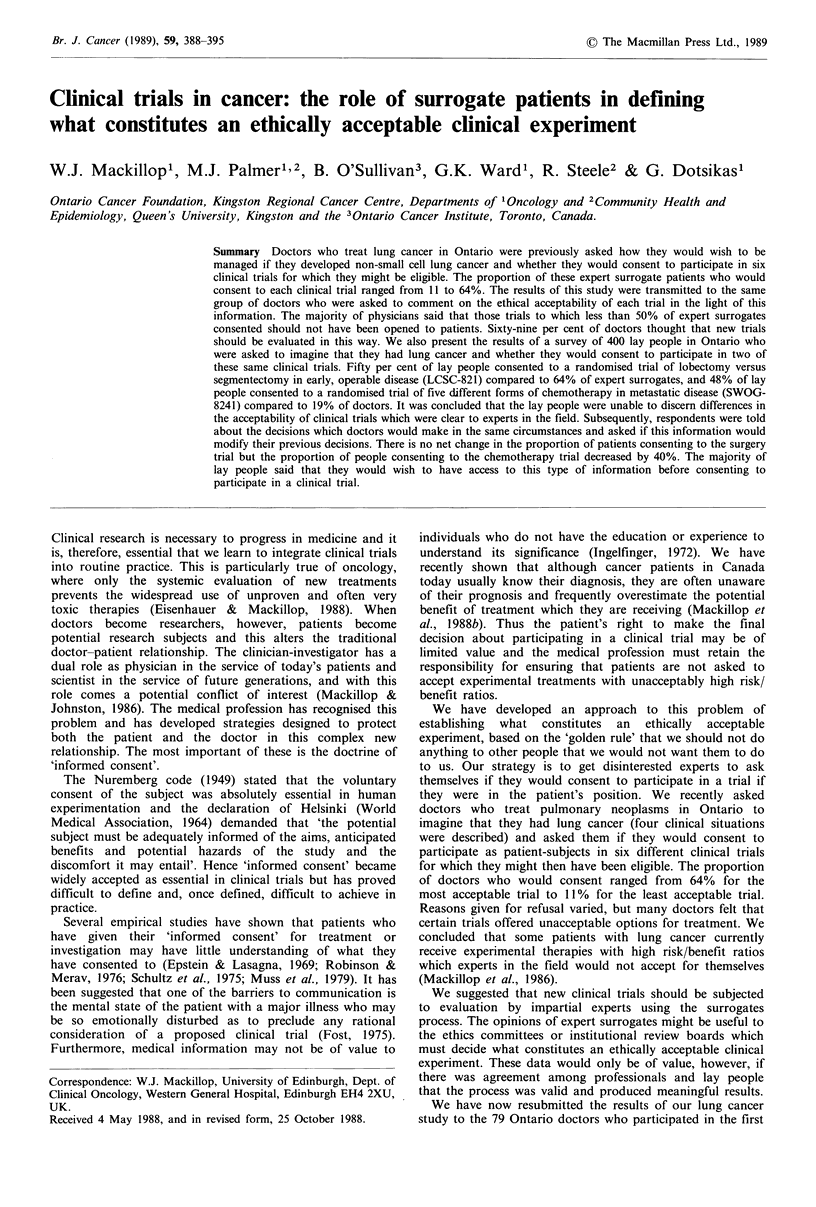

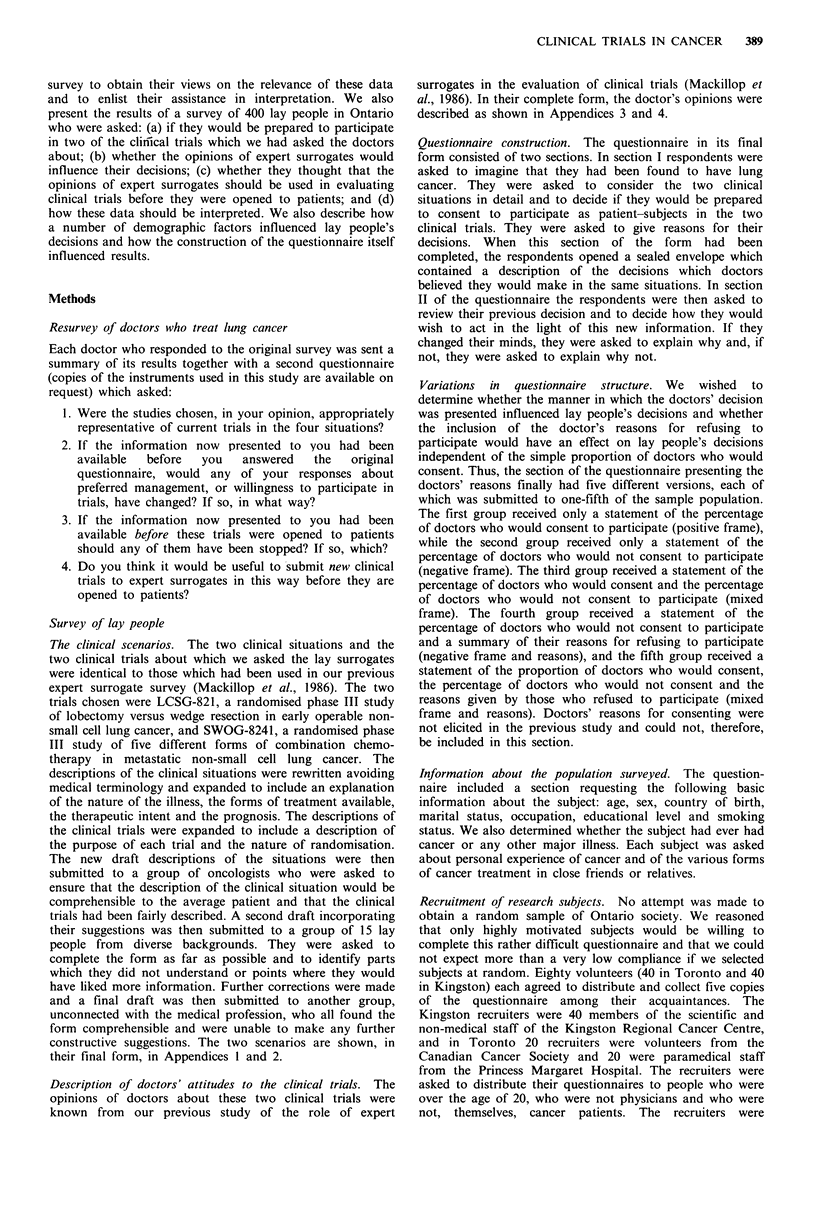

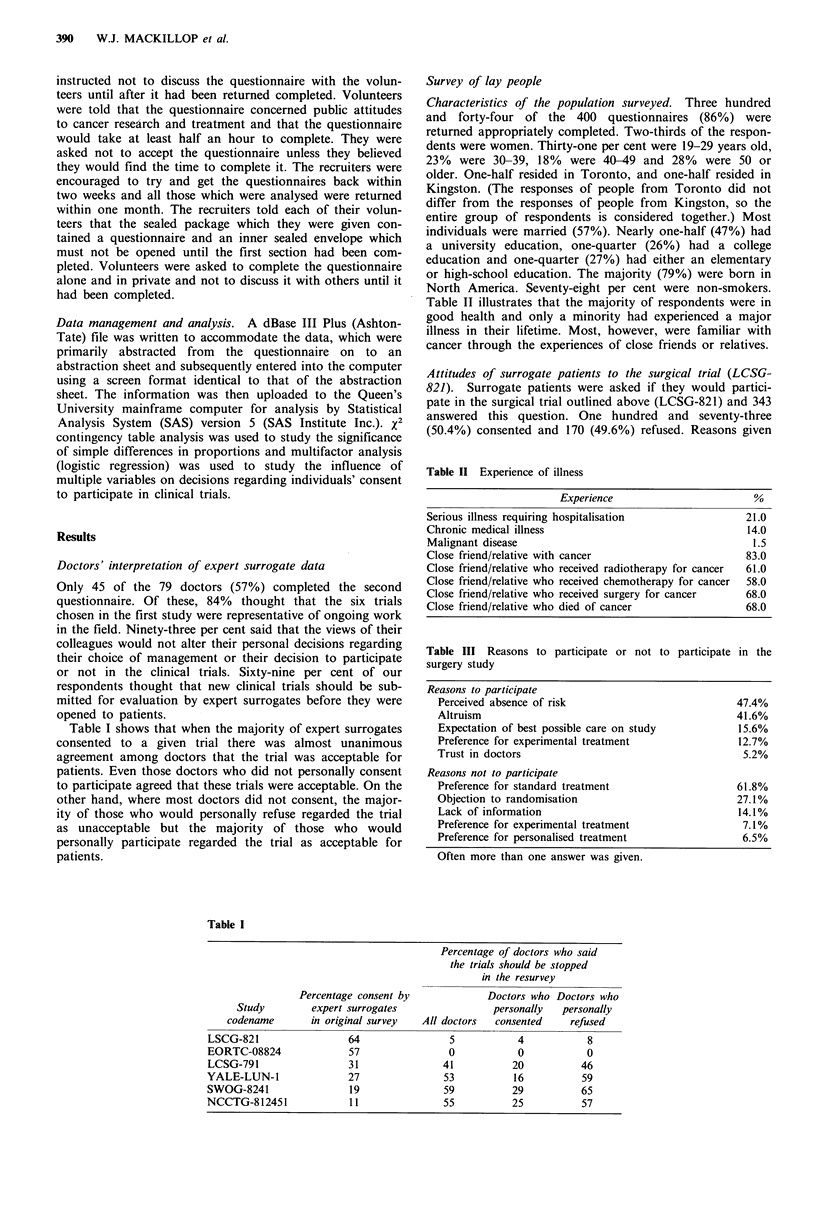

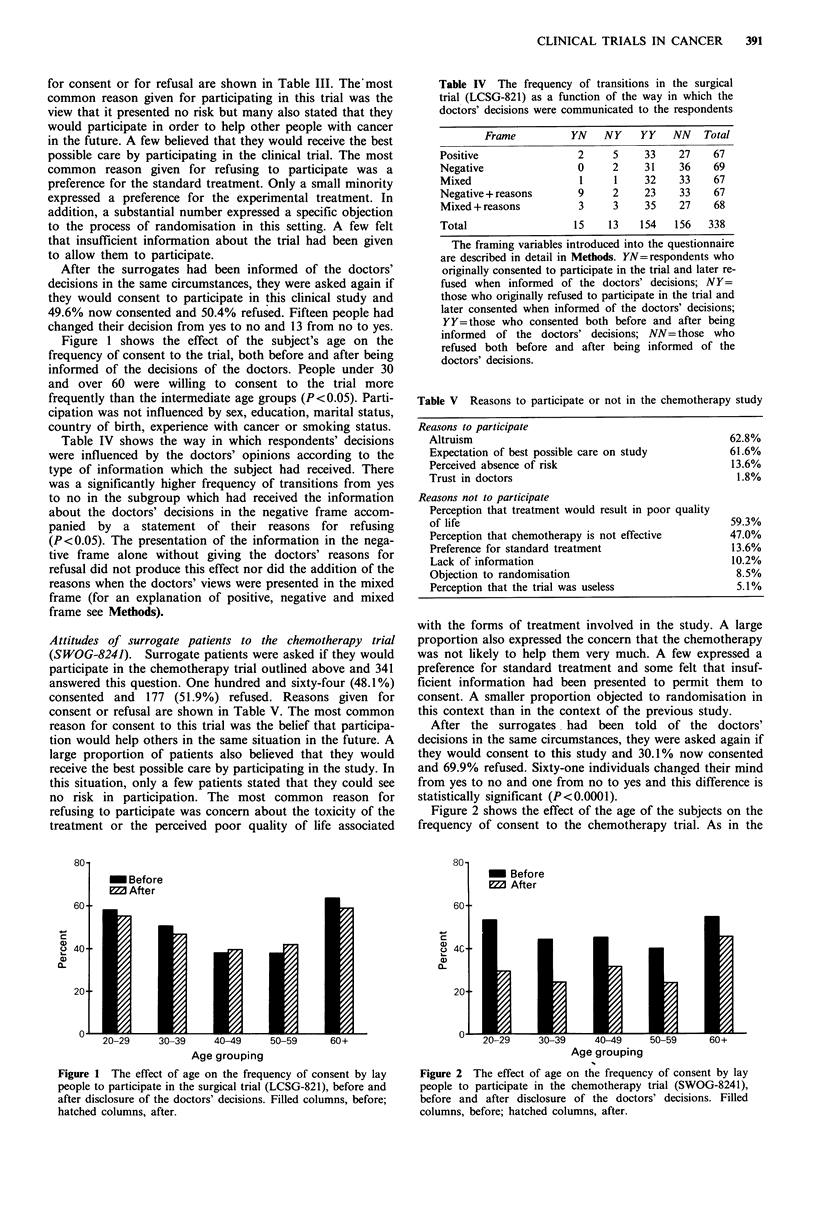

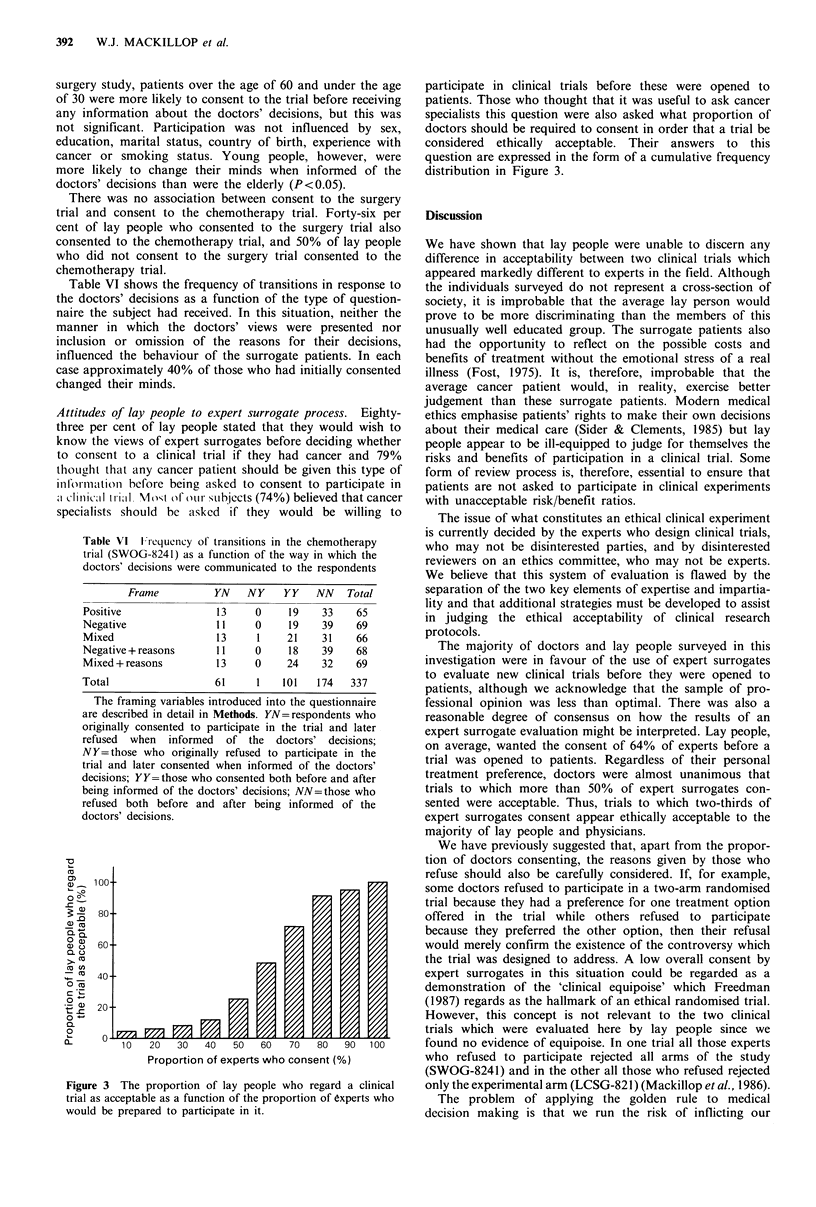

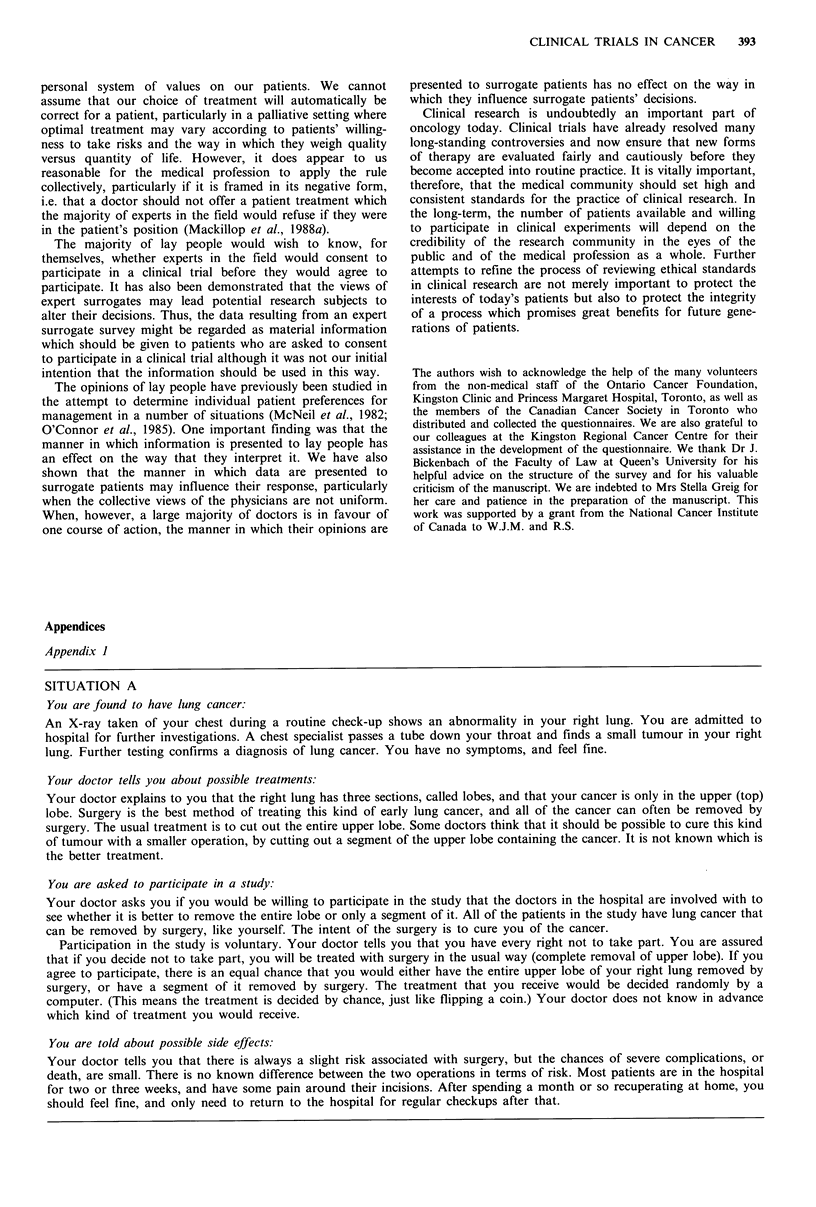

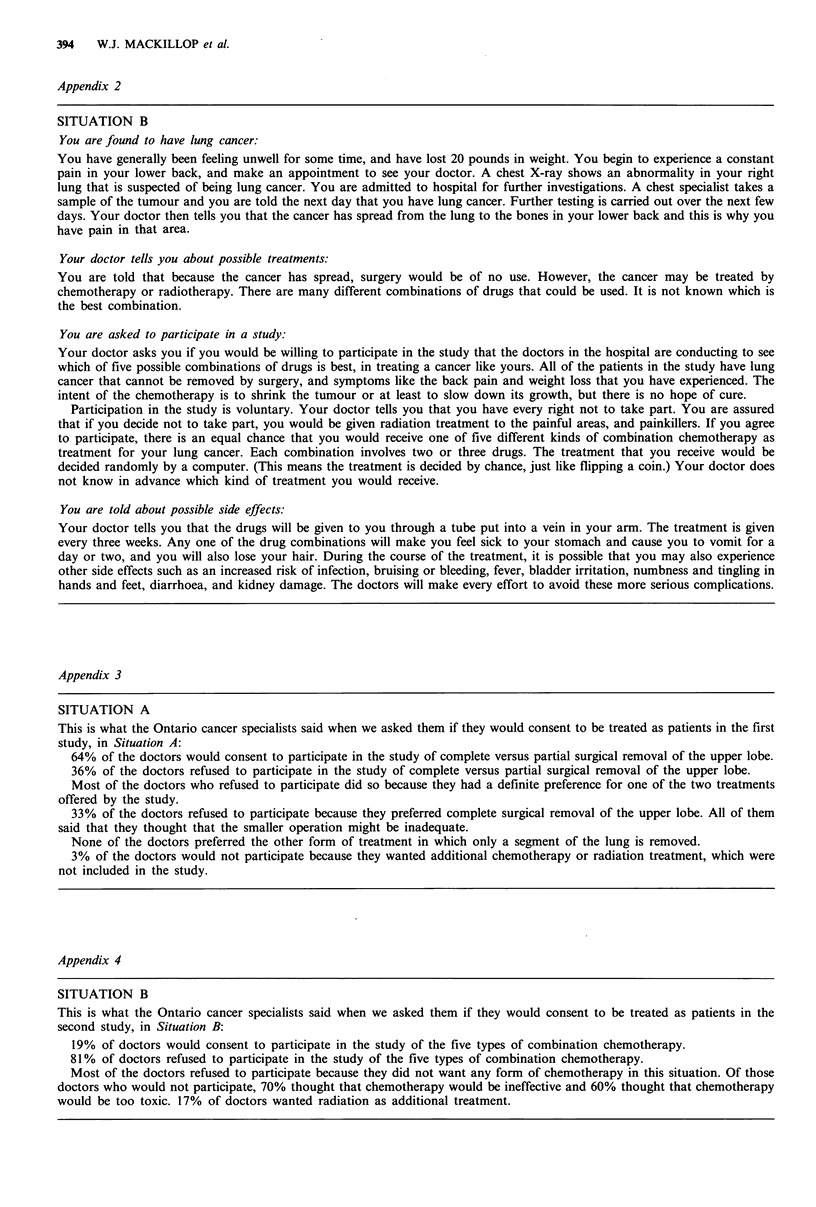

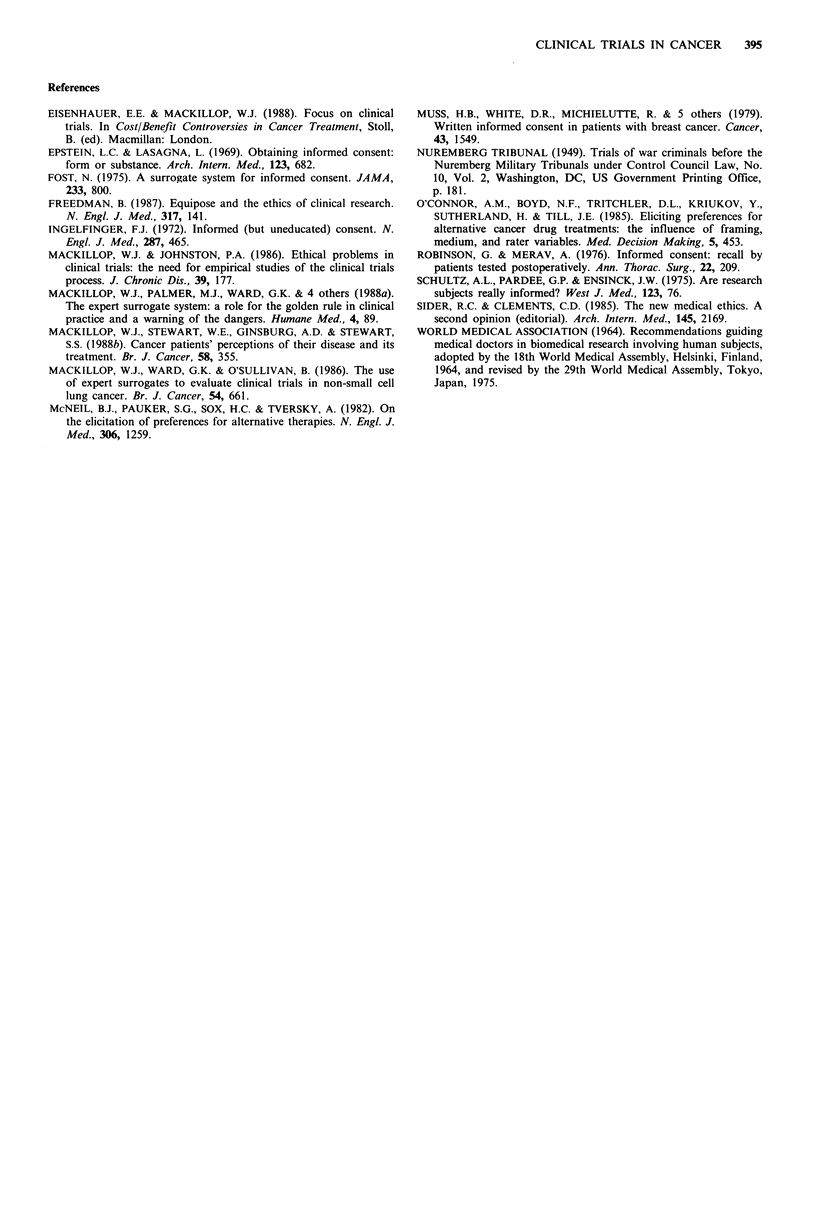


## References

[OCR_01001] Epstein L. C., Lasagna L. (1969). Obtaining informed consent. Form or substance.. Arch Intern Med.

[OCR_01005] Fost H. C. (1975). A surrogate system for informed consent.. JAMA.

[OCR_01009] Freedman B. (1987). Equipoise and the ethics of clinical research.. N Engl J Med.

[OCR_01013] Ingelfinger F. J. (1972). Informed (but uneducated) consent.. N Engl J Med.

[OCR_01017] Mackillop W. J., Johnston P. A. (1986). Ethical problems in clinical research: the need for empirical studies of the clinical trials process.. J Chronic Dis.

[OCR_01027] Mackillop W. J., Stewart W. E., Ginsburg A. D., Stewart S. S. (1988). Cancer patients' perceptions of their disease and its treatment.. Br J Cancer.

[OCR_01032] Mackillop W. J., Ward G. K., O'Sullivan B. (1986). The use of expert surrogates to evaluate clinical trials in non-small cell lung cancer.. Br J Cancer.

[OCR_01037] McNeil B. J., Pauker S. G., Sox H. C., Tversky A. (1982). On the elicitation of preferences for alternative therapies.. N Engl J Med.

[OCR_01042] Muss H. B., White D. R., Michielutte R., Richards F., Cooper M. R., Williams S., Stuart J. J., Spurr C. L. (1979). Written informed consent in patients with breast cancer.. Cancer.

[OCR_01053] O'Connor A. M., Boyd N. F., Tritchler D. L., Kriukov Y., Sutherland H., Till J. E. (1985). Eliciting preferences for alternative cancer drug treatments. The influence of framing, medium, and rater variables.. Med Decis Making.

[OCR_01059] Robinson G., Merav A. (1976). Informed consent: recall by patients tested postoperatively.. Ann Thorac Surg.

[OCR_01063] Schultz A. L., Pardee G. P., Ensinck J. W. (1975). Are research subjects really informed?. West J Med.

[OCR_01067] Sider R. C., Clements C. D. (1985). The new medical ethics. A second opinion.. Arch Intern Med.

